# A proposed unified interphase nucleus chromosome structure: Preliminary preponderance of evidence

**DOI:** 10.1073/pnas.2119101119

**Published:** 2022-06-24

**Authors:** John Sedat, Angus McDonald, Hu Cang, Joseph Lucas, Muthuvel Arigovindan, Zvi Kam, Cornelis Murre, Michael Elbaum

**Affiliations:** ^a^Department of Biochemistry and Biophysics, University of California, San Francisco, CA 94158;; ^b^Micron School of Materials Science & Engineering, Boise State University, Boise, ID 83725;; ^c^Salk Institute for Biological Studies, La Jolla, CA 92037;; ^d^Division of Biological Sciences, Department of Molecular Biology, University of California, San Diego, CA 92092;; ^e^Department of Electrical Engineering, Indian Institute of Science, 560012 Bengaluru, India;; ^f^Department of Molecular Cell Biology, Weizmann Institute of Sciences, 760001 Rehovot, Israel;; ^g^Department of Chemical and Biological Physics, Weizmann Institute of Sciences, 760001 Rehovot, Israel

**Keywords:** cryo-EM tomography, electron microscopy, deconvolution, chromosome structure

## Abstract

Cryoelectron tomography of the cell nucleus using scanning transmission electron microscopy and deconvolution processing technology has highlighted a large-scale, 100- to 300-nm interphase chromosome structure, which is present throughout the nucleus. This study further documents and analyzes these chromosome structures. The paper is divided into four parts: 1) evidence (preliminary) for a unified interphase chromosome structure; 2) a proposed unified interphase chromosome architecture; 3) organization as chromosome territories (e.g., fitting the 46 human chromosomes into a 10-μm-diameter nucleus); and 4) structure unification into a polytene chromosome architecture and lampbrush chromosomes. Finally, the paper concludes with a living light microscopy cell study showing that the G1 nucleus contains very similar structures throughout. The main finding is that this chromosome structure appears to coil the 11-nm nucleosome fiber into a defined hollow structure, analogous to a Slinky helical spring [https://en.wikipedia.org/wiki/Slinky; motif used in Bowerman *et al.*, *eLife* 10, e65587 (2021)]. This Slinky architecture can be used to build chromosome territories, extended to the polytene chromosome structure, as well as to the structure of lampbrush chromosomes.

## Significance

Cryopreservation of the nuclear interior allows a large-scale interphase chromosome structure—present throughout the nucleus—to be seen in its native state by electron tomography. This structure appears as a coiled chain of nucleosomes, wrapped like a Slinky toy. This coiled structure can be further used to explain the enigmatic architectures of polytene and lampbrush chromosomes. In addition, this new structure can further be organized as chromosome territories: for example, all 46 human interphase chromosomes easily fit into a 10-μm-diameter nucleus. Thus, interphase chromosomes can be unified into a flexibly defined structure.

A recent publication introduced iterative deconvolution for scanning transmission electron microscopy (TEM) tomograms of cryopreserved cellular structures (ref. 1 and references therein). Micron-thick areas of the vitrified cells were accessible without prior cryosectioning or lamella preparation. The deconvolution computation simplified interpretation of the tomograms by substantially filling the missing wedges of information that result from incomplete tilts. The effect was a substantial improvement in resolution along the depth (*Z*) direction. This technology made it possible to assess a tomogram from an area of the nucleus intact, in which large-scale interphase chromosome structures were noted (1).

Here, the chromosome structures observed in these nuclear tomograms are further documented and analyzed. This paper is divided into four parts. The first part presents the evidence, preliminary but compelling, for a unified interphase chromosome structure. The second part presents the proposed unified interphase chromosome architecture. The third part shows that this interphase chromosome structure could be further organized as chromosome territories: for example, by fitting the 46 human chromosomes into a 10-μm-diameter nucleus. The fourth part unifies this structure into a polytene chromosome architecture and lampbrush chromosomes. The paper concludes with a living light microscopy cell study showing that the G1 nucleus has very similar structures throughout this organelle.

The interphase nucleus encloses the genomic DNA, as well as the machinery for regulation of gene expression, RNA synthesis, and DNA replication (2). DNA is packaged into chromatin, the in vivo structure of which remains unclear. While mitotic chromosomes are highly condensed, interphase chromosomes decondense but remain in distinct territories with little overlap. Interphase chromatin is organized in a number of ways, including immutable gene-rich and -poor domains in the primary sequence and expression-promoting or -suppressing regions that may vary during the cell cycle or reflect cell differentiation (2). A classic distinction is drawn between euchromatin and heterochromatin, with the former more “open” and prone to expression, while the latter is more “closed” and prone to silencing (but see ref. 3). However, different methods, such as fluorescence and electron microscopy (EM), or posttranslational histone modifications, are sensitive to different parameters and do not necessarily agree in their identification.

The predominant model to describe the path of the DNA strand in the interphase nucleus is the constrained random walk (4) or fractal globule (5, 6). At prophase, the dispersed polymers must recondense without entangling. During mitosis, the space-filling interphase chromatin condenses into a compact micrometer-sized structure. The degree of order in these structures remains undefined.

The double-stranded DNA polymer itself, which in isolation appears as a semiflexible, right-hand helix 2 nm in diameter, winds tightly around core histones to form nucleosomes. Each nucleosome has a DNA footprint of 146 base pairs and a geometric diameter of ∼11 nm (7–9). Nucleosomes appear as beads on a string, but the density and spacing of nucleosomes along the DNA sequence may be highly variable (10). In the next stage, the nucleosomes are supposed to coil up into a 30-nm filament, possibly as a tight solenoid or alternatively with a zig-zag structure (11, 12). Today, the 30-nm filament is largely considered an artifact (13, 14). It has been observed in vitro, in isolated or ruptured nuclei, and in cases of deliberate manipulation of divalent cation concentration, but not in intact nuclei (13–15).

Current insights into chromatin structure arise primarily from methods based on sequencing. With a number of significant variations, chromatin is cross-linked, cleaved, captured, and sequenced in order to determine which sequences lie in close proximity (5, 16). These methods have revealed a genetic structure of chromosome territories at the largest scale, active and inactive compartments at the multimega base level, followed by topologically associated domains (also known as TADs) whose regulation is controlled concomitantly even if they appear to be distant in sequence (16, 17). An overall, three-dimensional (3D) spatial map of the genome can be generated from the proximity constraints. Extension of the methods to analysis of individual cells revealed a strong heterogeneity, however, making it difficult to connect proximity data to local structure.

Microscopy offers the most direct observations of structure, but specimen preparation may be disruptive. Classic EM requires fixation, followed by solvent-based dehydration and impregnation with a hardening polymer. Heavy metal salts are added to generate image contrast based on electron scattering; the indirect nature makes it difficult to interpret apparent density in terms of molecular composition (18). This limitation was circumvented by electron spectroscopic imaging, which distinguishes protein from nucleic acid on the basis of nitrogen and phosphorus concentrations (19). A recent advance used a DNA-binding dye to induce a localized polymerization of diaminobenzidine, which in turn binds an osmium stain (15). A modest density difference between euchromatin and heterochromatin was found, but no evidence was seen for long-range order (15). Fluorescence microscopy has made great advances with the introduction of superresolution methods. The combination with in situ hybridization permits even a degree of sequencing in situ. Still, long exposures and biochemical manipulations require strong cross-linking, which necessarily influences local structure. Cryoelectron tomography offers the most direct and pristine view of cellular structure, including chromatin, but conventional TEM requires thin sectioning or lamella fabrication using the focused ion beam microscope.

Cryoscanning transmission electron tomography is a new addition to the toolkit of cellular imaging techniques. The most obvious advantages in relation to conventional defocus phase-contrast TEM are the ability to accommodate thicker specimens and the quantitative contrast based on electron scattering cross-sections. As implemented for cellular tomography, it provides: 1) a unipolar optical transfer function with the specimen in focus, 2) a long depth of field, and most importantly, 3) strong contrast for low spatial frequencies (20). We have recently demonstrated the application of cryoscanning transmission electron tomography in combination with 3D iterative deconvolution processing to whole-cell tomography and obtained a view of the cell nucleus that revealed unexpected large-scale structures (1).

## Data Considerations and Their Complexity.

The primary data for this paper have considerably different attributes, such as the close-spaced 3D pixels with subtle gray level differences and textures, requiring new ways to display its details and architecture. This is a common problem for various imaging technologies (e.g., cellular cryoelectron tomography and MRI). The usual methods to visualize 3D data, such as moving up and down in the *Z*-dimension through two-dimensional (2D) slices, do not adequately show 3D relationships. Therefore, we propose the extensive use of stereo with extensions to circumvent this problem. Evidence for the chromosome structure is presented primarily in 3D stereo movies of various kinds (Movie S1 and rocking angular stereo pairs A to C′ presented in Movies S2–S8). Visualization guidance and challenges for the stereo movies are also discussed in depth in *SI Appendix*.

## Results Part I: The Preponderance of Experimental Evidence for a Defined Interphase Nuclear Chromosome Structure

### Deconvolved Cryoelectron Tomogram Nuclear Data: First Glimpse.

**Fig. 1.**
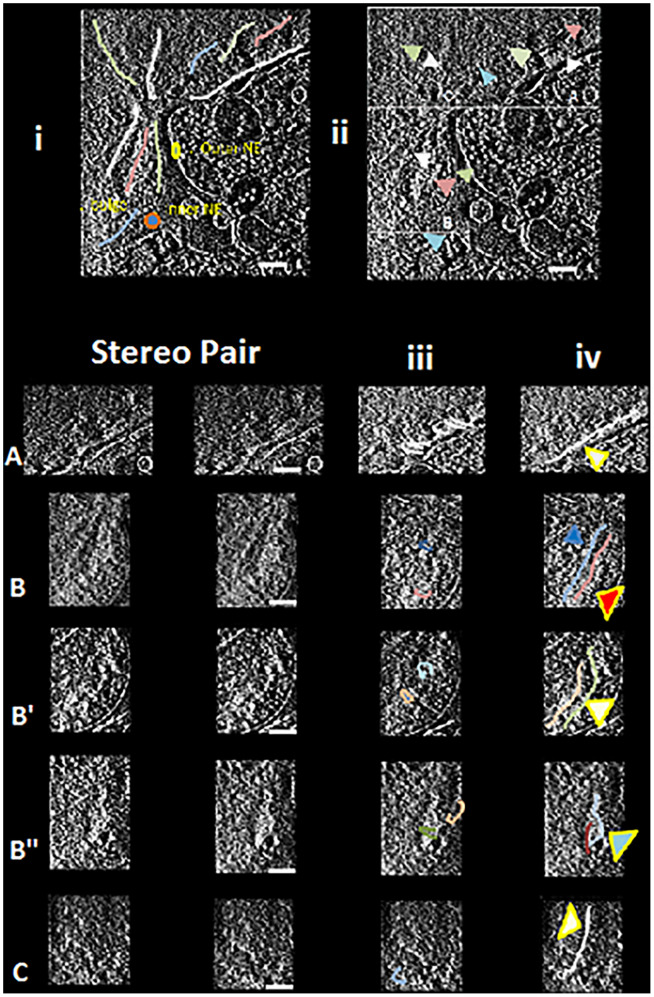
Large-scale banded structures are seen by deconvolution of the cryoelectron tomogram nucleus. This figure serves as a guide to visualize the large-scale banded chromosome structures seen in Movie S1 in a 3D data cube with the ability to move up and down the *z* axis (by moving the tab left/right). This tab is displaceable to prevent occlusion and coded to record the *Z*-position as described in the text. (*i*) A portion of the nuclear edge with large-scale structures highlighted with colored lines. (*ii*) Structures referenced in Movie S1 and Table 1 located in the nuclear boxes A to C. The left lower part represents stereo images (see below stereo guide) for the boxed nuclear regions, marked A–C at various *Z* nuclear depths (Table 1, *Z* column). (*iii*) Banded regions referenced in Table 2. Drawn band loops are shown. (*iv*) More examples of the large-scale structures and their bands. (Scale bars, 400 nm.) cyl., cylindrical; Euchr, euchromatin; int., internal; x-s, cross-section. Large-scale structured regions are drawn. See *SI Appendix* for the tutorials for visualizing stereo images by the cross-eyed technique.

The primary experimental evidence for a new interphase nuclear chromosome structure comes from a scanning TEM cryoelectron tomogram nuclear data that was deconvolved (1) and is shown in Movie S1; Fig. 1 contains a guide to approach this movie. The nucleus cryoelectron tomogram data originated from a cultured fibroblast cell line, prepared as described in detail in a recent publication (1). This nucleus shows a protrusion from the nucleus with distortions of the nuclear outer membranes (Movie S1), a known perturbation of the nucleus with nuclear lamin network perturbations (ref. 21 and references therein).

Movie S1 shows a 3D deconvoluted nucleus data cube with the ability to rapidly move up and down in the nucleus *Z*-plane by the quick bar movement (with a number on the bar that correlates to the *Z* position) (see legend to Fig. 1). The nucleus is divided into three box areas, A to C, to define the nuclear subregions. An additional description of this nucleus is shown in Fig. 1, *i* and *ii*.

In this nucleus, a large-scale structure—a structure with a variable size width of 100 to 300 nm and many microns in length—can be seen in multiple instances (highlighted in Fig. 1, *i* and *ii*). This structure bends and twists along its length as a thick rope-like structure and is present throughout the nucleus. Both the heterochromatin (next to the nuclear envelope) and euchromatin (in the interior) of the nucleus appeared to be built with the same structures. A study of the large-scale structure suggests that it is banded with transverse bands (100 to 300 nm), both for the heterochromatin and euchromatin. Table 1 highlights many of these banded large-scale structures at specific *Z*-planes, interpreting the large-scale structure positions. When carefully moving up and down in *Z*, the banded large-scale structures, which extend for long distances, stand out for each boxed area (A to C). These banded large-scale structures are further detailed in the stereo-pair movies.

**Table 1.** The *Z* position, location, and diameter dimensions for the large-scale structures shown in Fig. 1

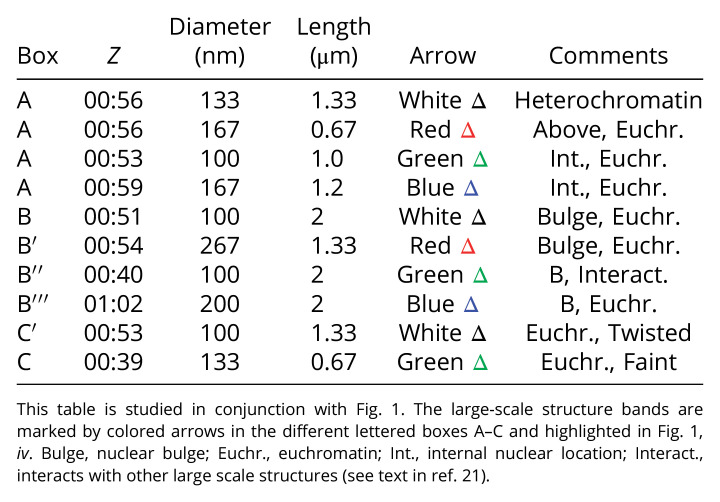

### Detailed Architecture of the Large-Scale Structure Banded Structures.

The rocking angular stereo-pair movies were used for the analysis of the large-scale structure bands. Fig. 1 presents the rocking angular stereo-pair movies corresponding to the nuclear boxes A–C at a precise *Z*-depth. The top two panels in Fig. 1 show lines for the large-scale structure to highlight distinct structures (see the figure legend).

A summary of the banded large-scale structures from the different nuclear regions from Fig. 1 is presented in Table 2. The lower right-hand panels of Fig. 1 have copies of the stereo pairs, with arrowheads, for the study of the rocking angular stereo-pair movies. The reader is advised to carefully study these rocking angular stereo-pair movies using the guidelines outlined in *SI Appendix*; though subtle, the structures can be discerned.

**Table 2.** Rocking angular stereo-pair movies (Movies S2–S8) used to study the large-scale structures and their bands

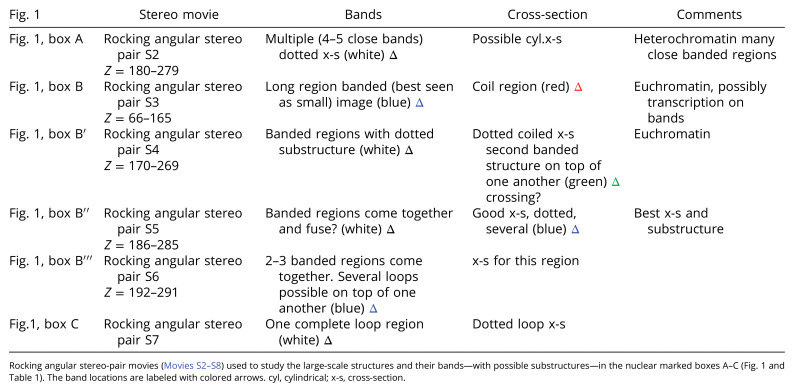

Nuclear box A is the first chromosome structure study, a region that contains largely heterochromatin structures, detailed in Fig. 1, and studied in rocking angular stereo-pair movie, Movie S2. The structural features are presented in Table 2. The heterochromatin large-scale structures (Fig. 1, arrowheads) show fine, punctuated, closely packed bands estimated to be ≤20-nm thick, inclined (the inclined band size/diameter measures ∼300 nm), and appear to be packed in phase with one another. The rims suggest relatively uniform-sized dots across many bands for many microns in length. One of the large, tilted views (Fig. 1, arrowheads) suggests possible cylindrical cross-sections of the heterochromatin bands.

Next, for the large-scale structures in the bulge region (largely euchromatin) of the nucleus, box B (and the different *Z*-depths B to B′′′) are described by studying the rocking angular stereo-pair movies. A large number of large-scale structures, many at different depths with lengths of many microns, enter this bulge. When moving down these large-scale structures, the transverse bands stand out. Various large-scale structures have densely packed banded transverse regions (see arrowheads in Fig. 1) with curving arcs or horseshoe/cylindrical structures. Some are broken up with thickenings at places in the arcs, possibly due to transcription events, making it difficult to complete the arc. In B′ and B′′, a particularly banded region can be highlighted (Fig. 1, arrowheads), with extensive dotted structures (suggestive of nucleosomes) following misshapen coiled bands. Note that many of the arc/horseshoe band structures are bent/twisted as they seem to coil around. In B′ and B′′, a dense banded region (Fig. 1, arrowheads) is seen, and is likely two or three large-scale structures on top of each other, suggesting a coiled region in the middle (Fig. 1, arrowhead). This region could potentially be a putative TAD structure (16, 17) but remains to be confirmed. Furthermore, there are regions where it is difficult to understand the architecture of the large-scale structures due to the severe fragmentation.

A complication in interpreting the large-scale structures and their banded loops is the presence of regions that seem to suggest two structures that are very similar to each other in bending and twisting, helically winding around each other. It is uncertain whether these phenomena reflect replication, homolog association (22), or other complications of chromosome structures.

Rocking angular stereo-pair Movie S7 (especially rocking angular stereo-pair Movie S8) has highlighted an almost complete coil of a dotted chain with a diameter of 200 to 300 nm (subject to the method of measurement) moving down a suggestive large-scale structure.

**Fig. 2.**
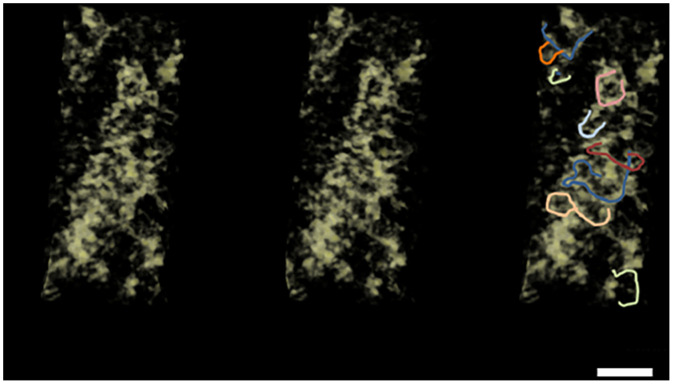
A representative stereo-pair, cut from nuclear box B, of a multibanded chromosome region. The possible distorted looped coiled structures (with its substructures) are shown. The third panel indicates drawn band loops. (Scale bar, 200 nm.) This figure was produced using UCSF Chimera software to make the stereo image (54). See *SI Appendix* for tutorials for perceiving stereo images using the cross-eyed technique.

A stereo pair (Fig. 2) at a somewhat higher magnification shows a representative series of band loops coiled about each other. The dotted coiled band structure, possible nucleosomes are also highlighted (Fig. 2, colored traces).

In summary, the entire interphase nucleus is composed of both closely packed large-scale structures with diameters of 100 to 300 nm and transversely banded (likely coiled) structures, in a rather regular defined architecture.

## Results Part II: The Proposed Interphase Chromosome Structure

Next, the large-scale structures and their transverse bands were integrated into a molecular structure. A software package was used, described in the *Materials and Methods*, to quantitatively model multiple sequential levels of helical coiling as a structure. The dimensions were tracked and scaled accurately at all levels of the structure. Once the structures are built, they can be analyzed in terms of either orientation, size, or dimension.

**Fig. 3.**
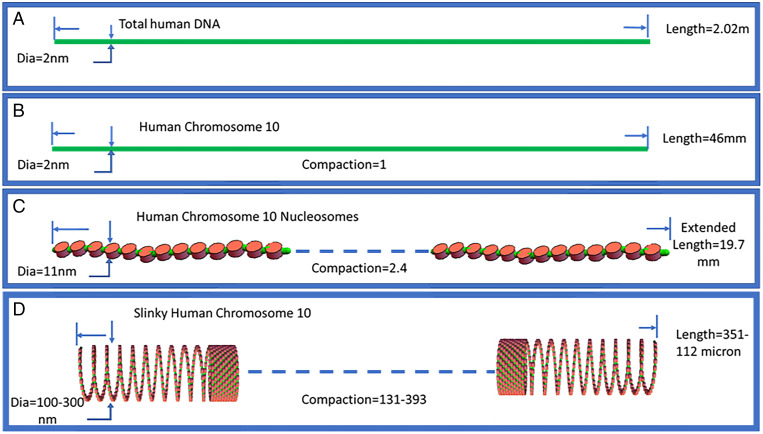
Depiction of different degrees of organization of DNA in the interphase nucleus. (*A*) Total human DNA. (*B*) Human chromosome 10 as a representative chromosome. (*C*) Human chromosome 10 is organized as a 11-nm nucleosome fiber. (*D*) The nucleosome fiber for human chromosome 10 is then coiled as a Slinky with a representative diameter of 200 nm. The maximal extension of the NS fiber was [669 K nucleosomes × (11 nm [nucleosome] + 18.4 nm [the extended linker DNA])], while the compact nucleosome fiber is [669 K nucleosomes × (11 nm [nucleosome] + (2 × 2 nm [DNA] for hairpin bent linker))]. The compaction is DNA length/Slinky length, where the Slinky length is [(10 mm, the compressed nucleosome fiber length [see text])/Slinky gyrus circumference (to give the number of Slinky gyri)] × 11 nm (the gyrus size assuming maximal Slinky packing). See text for further details.

**Fig. 4.**
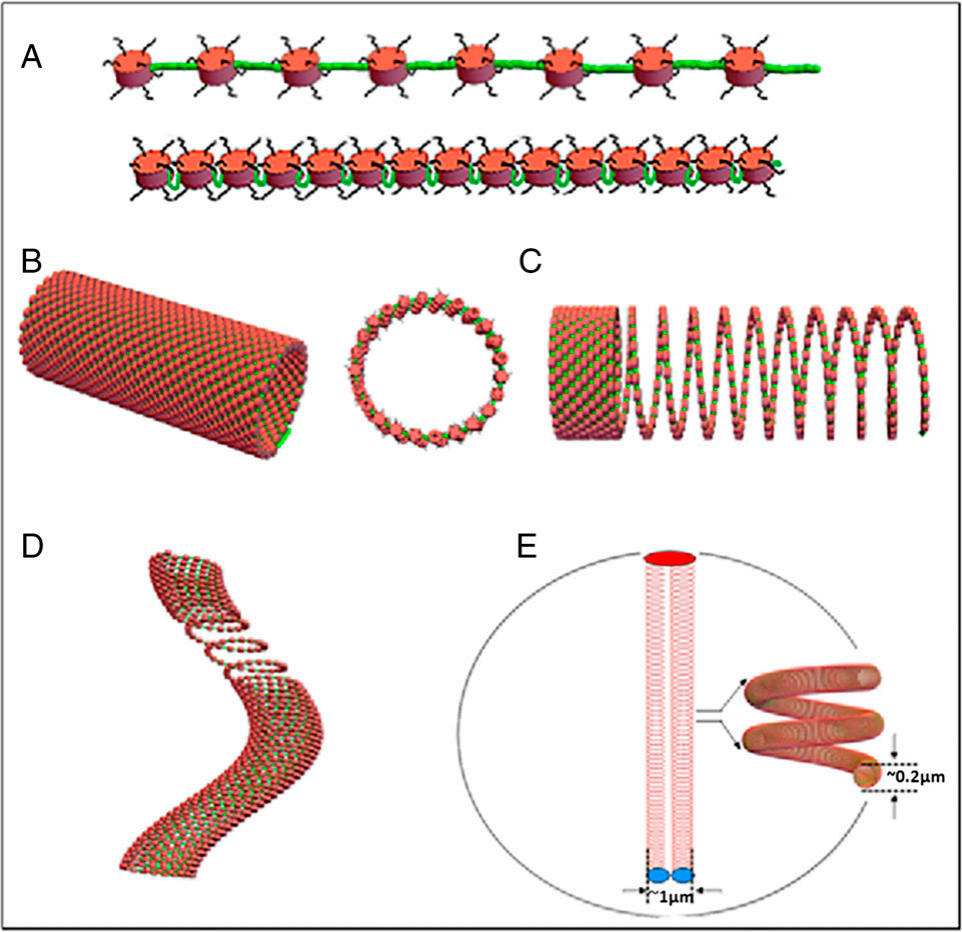
The Slinky as a chromosomal structure motif to explain large-scale structures and its bands. (*A*) Nucleosomes, to scale, with green linker DNA maximally extending the nucleosomes. The bottom panel shows nucleosomes compressed with the linkers looped out, like a hairpin; the looped linkers could act as a molecular antenna with special sequences facing out of the hairpin. (*B*) The coiled nucleosomes (with a diameter of 100 nm in this case) as a Slinky. The middle Slinky structure shows a hollow cross-section (diameter = 100 nm), while a Slinky diameter of 200 nm with maximally condensed nucleosomes would have ∼63 nucleosomes per Slinky gyrus. (*C*) Slinky as compressed as possible (for heterochromatin) or extended (for the transcribed regions). (*D*) A bent Slinky structure with an extended active transcribed region. (*E*) Human chromosome 10, a metacentric chromosome, coiled as a Slinky, but organized in a 10-μm diameter nucleus (drawn to scale). The Slinky requires extensive bending and twisting of the Slinky structure (shown in the nucleus *Inset*) to fit in the nucleus in the Rabl configuration (26, 27).

A quantitative perspective of this proposal is described in Fig. 3. First, human diploid DNA, based on sequence, has a total length of 2.02 m (Fig. 3*A*). A representative human chromosome 10 would have a DNA length of 46 mm, 2 nm in diameter, and its compaction is defined as 1 (Fig. 3*B*). Essentially, all DNA, on average, is organized as nucleosomes (200 base pairs per nucleosome), whereas the human chromosome 10 nucleosome chain (if fully extended) would have a shortened length of 19.7 mm (Fig. 3*C*). This further compaction, when organized as a well-known 11-nm nucleosome fiber, would reduce the DNA length to ∼43% (Figs. 3 and 4). The nucleosome fiber can be fully extended (extended linkers) or compressed with looped linker sequences as a hairpin (Fig. 4*A*).

We propose that this 11-nm nucleosome fiber is further tightly coiled as a 100- to 300-nm-diameter structure (average diameter = 200 nm) whose diameter is the large-scale structure and the transverse bands the coils of the nucleosomes (Fig. 3*D*). This tightly coiled structure is analogous to a toy known as the Slinky (ref. 23; motif introduced in ref. 24). The Slinky has many attributes, and represents the structure proposed for the interphase chromosome architecture. This structure further compacts the DNA by ratios of 1:131 to 1:393 in length (depending on how tight the nucleosomes are packed into the Slinky bands and the diameter). The Slinky structure length (e.g., for human chromosome 10) ranges from 351 μm to 112 µm (Figs. 3 and 4). Prophase and mitotic chromosome structures are proposed using the Slinky architecture in a companion paper (25).

The Slinky structure is shown in Fig. 4. Fig. 4*A* shows the 11-nm nucleosome fiber, to scale, with the nucleosome linker regions extended. Alternatively, these linker regions can be looped out as molecular antennas, in which case the 11-nm nucleosome fiber is more compressed. Fig. 4*B* illustrates how the nucleosome fiber is coiled as a Slinky that can be regularly packed. Each gyrus could carry between 24 and 63 nucleosomes, depending on the gyrus diameter and nucleosome density. The corresponding lengths of DNA are 4.8 kb to 12.6 kb, corresponding to proteins encoding 1,600 to 4,200 amino acids. If the Slinky gyri are closely packed, this could represent heterochromatin. The interior of a cross-section is hollow, allowing for the interior and exterior to be largely topologically equivalent for free diffusion of transcription machinery and DNA replication events. Note that histone tails can be spatially extended to the interior and exterior. Fig. 4 *C* and *D* show how the Slinky can be extended for transcription (ranging from minor to major extension), depending on the degree of transcription. Indeed, one or more Slinky gyri can be completely extended as a looped structure. The extended Slinky structures can be bounded by more closely/densely packed Slinky regions. The elegance of the Slinky structure is that it is very flexible and can be easily distorted, such as squashing the Slinky to flatten, slanting the gyri for packing and bending, or bending/distortions of the looped-out Slinky regions during transcription events.

## Results Part III: Global Nuclear Architecture; Chromosome Territories

The Slinky structure must fit into a 5- to 10+-μm nucleus. For example, Fig. 4*E* shows human chromosome 10 to scale, fitting in a 10-μm nucleus. Human chromosome 10, as an average-sized human chromosome, is metacentric, meaning it divides the chromosome into two arms. However, many interphase chromosomes are organized such that the centromere is located on the nuclear envelope at one end of the nucleus, while the telomere end is at the opposite nucleus pole, according to the well-known Rabl configuration (26, 27). The Slinky length for human chromosome 10 (Fig. 4*E*), arranged in the Rabl configuration, indicates that some local bending/twisting/folding of the Slinky structure must be made to accommodate this length in the nucleus, as shown in Fig. 4*E*. In a real nucleus, this further coiling is likely to occur as irregular bending and twisting, although retaining the Rabl configuration. To accommodate for this twisting, 1∼2 μm can be allocated as potential space occupied by the chromosome, as the 45 other chromosomes in the Slinky structure also need to be considered. Nevertheless, the genesis of chromosome territory (28) is evident. The question remains whether the Slinky structure fits comfortably into an average-sized 10-μm nucleus diameter. If 2 m of human genomic DNA is maximally coiled into a 200-nm diameter Slinky (1/266th the DNA length), it would take up 25% of the nuclear volume. This leaves room for Slinky gyri expansion during transcription. This fraction of the Slinky volume relative to the nuclear volume decreased rapidly as the nuclear diameter increased. Nevertheless, the DNA in a Slinky structure could be closely packed, which is what one observes in live-imaging microscopy (see, for example, Fig. 6).

The next question is whether all 46 human interphase chromosomes in a Slinky structure (compressed as a chromosome territory example shown in Fig. 4*E*) fit within an average 10-μm-sized nucleus. A simple calculation, using the average length of human chromosome 10 as an example (and assuming all 46 chromosomes have this volume), indicates that the cylindrical representation (1-µm diameter × 10-µm length) gives a total volume for all 46 chromosomes of 361 µm^3^. This is comparable to a 10-μm-diameter nucleus volume of 524 µm^3^, leaving one-quarter of the nuclear volume free. Thus, this model of chromosome territory is plausible.

## Results Part IV: Extensions to the Unified Interphase Chromosome Structure

### A Polytene Chromosome Interphase Chromosome Example: Unified Interphase Chromosome Structure.

**Fig. 5.**
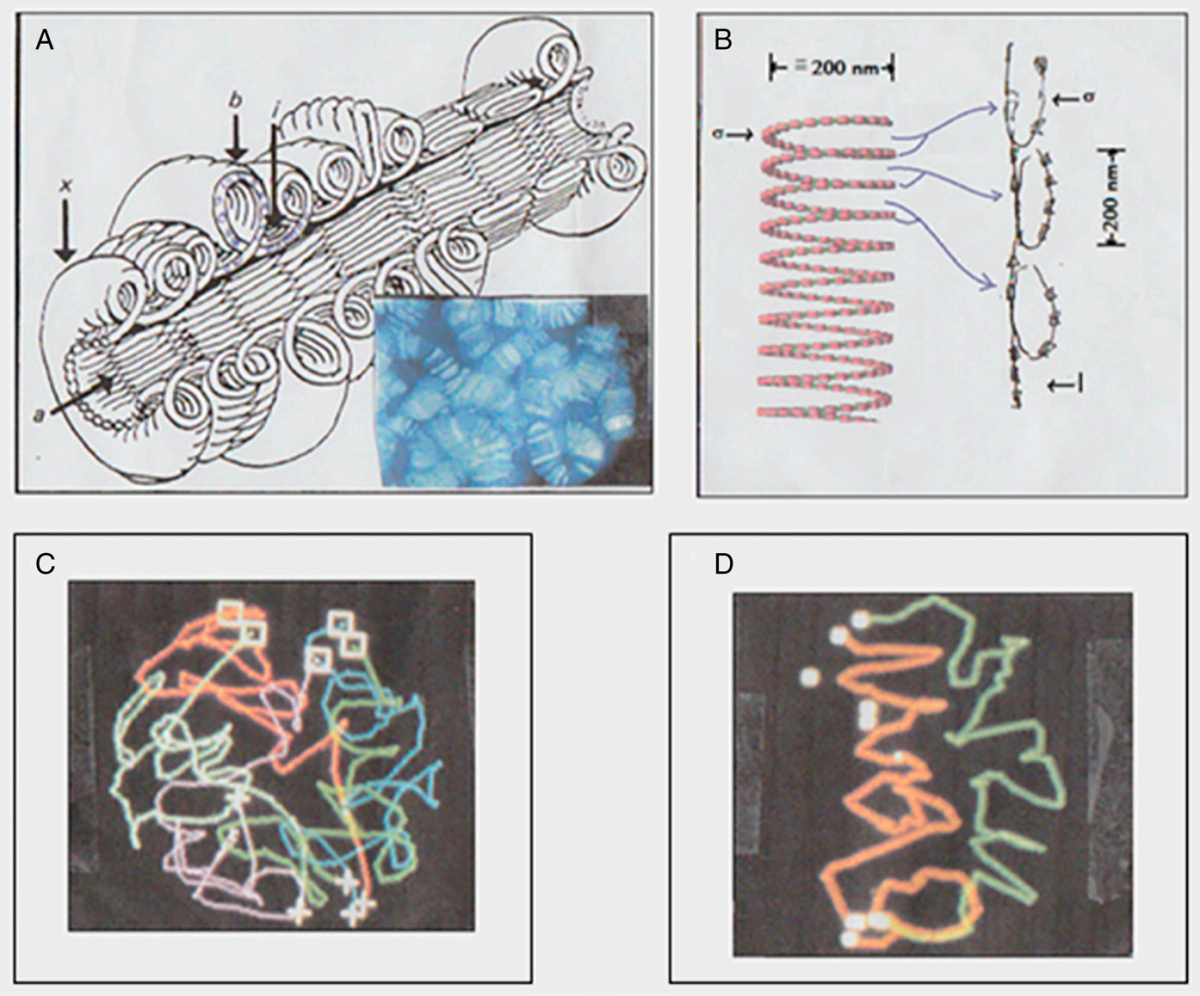
Polytene chromosome architecture can be unified with the interphase chromosome Slinky structure. (*A*) The polytene chromosome architecture is taken from figure 18 in Mortin and Sedat (30), and reproduced/adapted with permission. The *Inset* of the polytenes in the nucleus was taken from the J.S. archive. (*B*) Each polytene band is possibly a Slinky gyrus (≅200 nm in diameter) twisted 90° as shown, given the approximate dimensions of the polytene bands. (*C*) The polytene banded chromosomes, in a whole nucleus, can be determined, as shown in a Rabl configuration. Reproduced/adapted from figure 3 in Gruenbaum et al. (55) with permission. (*D*) Two adjacent polytene chromosomes from a 3D nucleus, as chromosome territories—never mixing with each other—are seen as right-handed sloppy-coiled structures (31); also in Rabl configurations. Data were taken from the J.S. archive.

One interesting feature of the Slinky chromosome structure is that it can unify other interphase chromosome structures. One of these interphase chromosomes with unsolved architecture is the polytene chromosome organization, seen in many insects and plants (29). These cells/nuclei are locked in G1/S such that the DNA replicates many times; however, the cells do not divide, resulting in large banded (∼200-nm band thickness) chromosome structures (29) (Fig. 5).

Fig. 5*A* shows the proposed polytene structure, extensively documented in a 1982 article (30). The 1,024 DNA copies were confined to a chromosome rim: a torus-radially symmetric chromosome architecture with a hollow interior. EM studies showed that the path of the DNA (as the fiber was undefined back then) is likely a single loop, ∼200 nm per side, corresponding to the band thickness (shown in Fig. 5*A*) (30). It is inferred that the fiber today is likely an 11-nm nucleosome fiber. We propose that the single loop is a Slinky gyrus of the same size, rotated 90° (as shown in Fig. 5*B*). The polytene band thickness is thus the same as that of the Slinky turn. Once the loop is rotated, it replicates radially with the same size, and the polytene band grows in width (3 μm for *Drosophila* salivary gland polytenes) as replication rounds proceed. For completeness, Fig. 5*C* shows a 3D polytene structure (31), with Fig. 5*D* indicating two side-by-side polytene chromosomes, right-handed helically coiled, in the Rabl configuration (27). Thus, it is possible to unify previously unsolved, diverse interphase chromosome architectures into a common Slinky molecularly identical structure.

### Lampbrush Chromosome Structure Can Be Integrated into the Slinky Architecture.

A lampbrush chromosome structure has always been difficult to integrate into the general interphase chromosome architecture. This enigmatic diplotene meiotic prophase I stage, with paired half-bivalents (two sister chromatids), has extensive transcriptionally active loops bounded by dense chromatin regions (refs. 32 and 33 and references therein). Nevertheless, this structure can be readily integrated into a Slinky architecture. We propose that the large transcriptionally active loops are simply looped-out single (or several) Slinky helical gyri, where the loop bases are confined, adjacent to more condensed Slinky helical gyri (like the proposed more silent heterochromatin Slinky regions). Transcriptional looping is a natural feature of the Slinky architecture as transcription first extends the Slinky gyri, further with more transcription, with the extension limit being a large loop. Thus, the Slinky architecture unifies the interphase chromosome structure.

### A Confirming Living Light Microscope Cell Study.

**Fig. 6.**
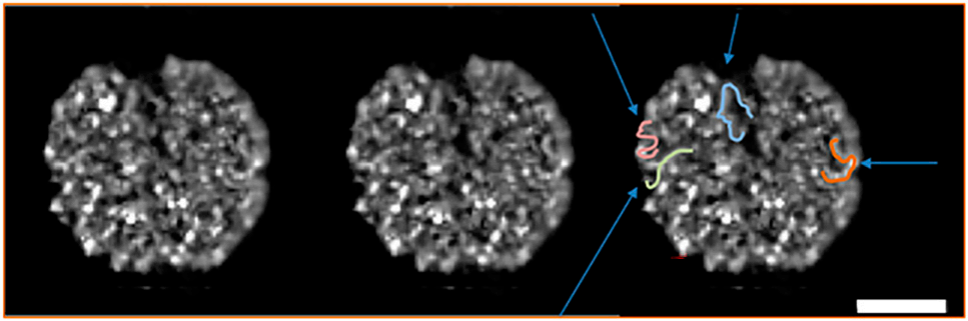
Live optical imaging at extremely low-excitation light intensity and deconvolution showing unique interphase chromosome structures. The first two images are a stereo pair of a 3D G1 cell nucleus, visualized by cross-eyed stereo technology. The third image is a duplication of one of the stereo pair, but blue arrows show the locations of some large-scale looped structures, and drawn chromosome looped structures as examples. (Scale bar, 4 μm.) See *SI Appendix* tutorial for stereo visualization technique.

The last piece of evidence for a specific interphase nuclear chromosome structure comes from live imaging of a B cell in a likely G1 cell-cycle state (34). The dominant problem in live imaging is phototoxicity from the photons used to excite specific fluorescence, giving rise to free radicals (35, 36), and subsequently the repair of the photo-damage that complicates the interpretation of the resulting image. To avoid photo-damage, the conventional excitation intensity is reduced by three to four logs: 1/1,000∼1/10,000 the usual intensity/dose of photons. The resulting images, taken as a four-dimensional (4D) data stack, are extremely noisy (*Materials and Methods*) and require computer processing with the newly developed entropy regularized deconvolution algorithm (37) to recover the image, as shown in Fig. 6 and as described in *Materials and Methods*. The interphase nucleus is fluorescently labeled, making the chromosomes visible (see discussion in *Materials and Methods*), but best studied as a stereo pair (Fig. 6). A study of the chromosome structure in the nucleus suggests that the entire nucleus is filled with a relatively uniform thick looping rope-like structure with a diameter of ∼260 nm (compared to the size bar). The thick fiber, pointed out by arrows in many places, has small variations or protuberances along their entire length. The fiber makes rapid bends and twists, and is densely packed, making close contact with other fibers approximately one or two fiber diameters away. The fibers tend to touch one another in multiple places.

In summary, evidence from living cells with minimal perturbations suggests that a G1 interphase chromosome structure is a thick, relatively uniform-diameter fiber. The live nucleus study presented here is suggestive, but the majority of the evidence for an interphase chromosome structure is founded by the cryoelectron tomographic study.

## Discussion

We have made the case for the existence of a unified interphase chromosome structure, denoted the Slinky, throughout the interphase nucleus. The key to the analysis leading to this structure was the visualization techniques of the cryoelectron tomographic deconvolution data. A few points to emphasize the known issues and features can be made.

First, this is a defined structure: an architecture using coiling as a structural principle. The Slinky structure, with its possible intrinsic order (Fig. 3*D*), lends itself for further protein–protein interactions; this is similar to a crystalline structure whose underlying architecture can undergo further modifications. Parts of the Slinky structure are so exposed that it may be considered a molecular antenna. To put this in perspective, no studies have found in vivo evidence for the 30-nm nucleosome structure (11, 12)—an in vitro structure—in living cells (13, 14). No statements regarding coil handedness were made, although this feature could be differentially used.

The question remains how the Slinky structures are built or maintained. While no explicit statement can be made, the polytene literature suggests that each polytene chromosome band may have specific proteins to individualize these structures (29), which may be characteristic of interphase Slinky as well. The cohesin complex, by its ring structure, has the ability to bring two structures together (38–40) for binding, and brings two adjacent Slinky gyri together, thus further organizing the Slinky. Furthermore, condensins can change the Slinky architecture (41, 42). Nevertheless, there is a great deal of flexibility and room for genetic modification; the Slinky gyri do have substantial size variation, which could have genetic ramifications (e.g., for genetic control/distinction). We are reminded that polytene chromosome bands have distinct thickness, size, and appearance variations (genetic location band maps) likely under genetic control (29). Recent literature documents the progress linking the *Drosophila* sequence information with genetic studies, and strongly suggest that the polytene band organization (bands, specific bands, and interbands) are highly correlated with the molecular and sequence information (43–45). Unfortunately, we cannot comment on where promoters, enhancers, or protein-coding sequences are located relative to the Slinky gyri, although these features could be coupled into the Slinky organization for further genetic specificity.

Second, there is a great deal of variation in the appearance of the Slinky bands: bands are bent, twisted, and misshapen, which is not surprising because of the solution and motion forces that occur in the nucleus. Thus, metabolic issues, transcription, and replication with their plethora of protein complexes must significantly change the Slinky structure, which can be inferred from the experimental data.

Third, the Slinky structure is very flexible and easily bent and moved around. However, whether the resulting polymer solution is following polymer statistics is unclear. Alternatively, the Slinky structures themselves moved around to specified locations maintaining long-range interactions. Indeed, from the data, the packing density for the large-scale structure is high, although large-scale structures were shown to be on top of each other in certain cases, possibly reflecting the familiar TADs (16, 17). Tangles, knots, or complicated looping configurations were not observed, as discussed in the literature (46, 47). The large-scale structure is also seen to be smoothly aligned and associating with one another.

Fourth, the Slinky structure is a delicate structure that is easily distorted and modified by chemical fixation, raising issues with the conclusions of these procedures.

Fifth, while there is a preponderance of evidence, both live optical data and the cryoelectron tomographic studies, leading to a defined chromosome structure, there is a great deal of unknowns allowing additional structures to be superimposed. Additional variants may be seen in different parts of the cell cycle. The poor definition of the cell cycle point for the data presented here may raise some questions regarding structural variations.

Finally, the major strength for the proposed structure in the light of so many existing models described in the literature (48, 49), is that our inferences were drawn from a cryopreserved vitrified aqueous environment, which preserved the likely living structural state.

In summary, preliminary evidence from cryoelectron tomographic nucleus data showed that the whole nucleus seems to be organized into a defined, ∼200-nm-diameter, coiled nucleosome interphase chromosome architecture, a structure similar to that of a Slinky. This structure also matched the live optical microscopy study, showing very similar image features and nuclear chromosome packing, as seen in the results. This flexible structure allowed a diverse interphase chromosome state, consisting of either the polytene chromosome state or lampbrush chromosomes, to be unified and understood as essentially part of the same organization. Thus, all interphase chromosomes may fundamentally have an identical underlying molecular architecture.

## Materials and Methods

### Living Nucleus Imaging Study.

The cells were prepared as described previously (34). Briefly, pro-B cells were purified from the bone marrow using B220 microbeads (Miltenyi Biotech) from mice carrying a tandem repeat of TET repressor elements in both alleles of the immunoglobulin heavy-chain locus. Isolated pro-B cells were grown for 5 d in the presence of interleukin-7 and stem-cell factor at 37 °C under 5% CO_2_ conditions. Actively growing pro-B cells were transduced with a virus expressing TetR-EGFP. Two days posttransduction, cells were imaged using the OMX v3.20.3537.0 microscope platform (Applied Precision). Typically, cells can be distinguished in G1 (early S) from later cell cycle stages by the presence of one or two Tet dots. Images were acquired using a 100×/NA 1.4 objective using 100 time points (each time point for 10 ms, 1 s per data cube), 0.15-μm *Z*-step (typically 30 to 50 *Z*-sections per cube), 2 OD/0.01 intensity attenuation, and a 5-ms exposure time. Even with the EMCCD (with high gain) as camera image acquisition, the individual *Z*-sections before deconvolution, showed only noise; *Z*-projections of the data could faintly see that a nucleus was present.

There are some qualifications for the study of live nucleus chromosomes. First, we note that the G1 cell cycle state was approximate. Second, the fluorescent chromosome label comes from low-affinity interactions involving TET-GFP repressor binding across the genome. Third, it will be important in the future to examine TET-GFP–mediated fluorescence across chromosomes using a range of intensities. Finally, we emphasize that this live chromosome dataset is merely a supporting example of the cryoelectron tomography data, which is the majority of evidence for the Slinky structure proposed in this study.

The deconvolution of the 3D or 4D light micrographs shown in Fig. 5 were obtained with ERDecon-II, according to Arigovindan et al. (37), but extended/updated by M.A. and Eric Brandlund, and then analyzed in PRIISM (see below). The deconvolution followed the previous publication but, in some cases, an additional time (second derivative) filter was used. In ERDecon-II, the time filter was a natural extension to the deconvolution software, built into the mathematics of ERDecon-II. The improvement of the 4D over the 3D deconvolution was slight, with Fig. 6 resembling the 3D version.

### Initial DC Processing.

The data for this study were obtained from an earlier publication (1). The cells were grown, plunged frozen, and processed for cryoelectron tomogram, as described previously. The tomograms were subjected to deconvolution and an initial representative nuclear deconvoluted cryoelectron tomogram image was presented.

The nucleus cryoelectron tomogram was further processed and analyzed at the University of California, San Francisco (UCSF). First, the deconvoluted data were intensity-scaled to remove (truncate) the pathological, sparse, isolated, few, extremely high-intensity pixels from the deconvoluted data (*SI Appendix*). Next, the deconvoluted image was histogram-trimmed, a process specific to deconvoluted images. The deconvoluted intensity histogram typically has a lower intensity value (mostly noise and poor deconvolution), while the upper intensity values, the broad higher intensity tail, has all (most) of the deconvoluted image/structural features (improved deconvolution, prominently filling the missing wedges) (1, 50). In addition, there were large intensity values originating from the calcium phosphate granules in the mitochondria. The low- and high-intensity values were then truncated. This process is discussed in detail in *SI Appendix*.

The histogram trimmed deconvoluted image was displayed in three dimensions using Quick-Time v7.7.9 display software (Movie S1). *Z*-planes can be studied (by moving up and down *Z* in the cube), selected by the movement of the cursor bar. The majority of the data for the interphase chromosome structure comes from rocking angular stereo-pair movies, described in detail in *SI Appendix* (some examples can be seen in ref. 50). The rocking angular stereo-pair movies allow 3D views of dense cellular structures for a detailed study and analysis. The computer scripts (written in PRIISM, see below) are available from the authors (J.S. and A.M.).

### Computer Modeling Software.

The computer modeling of the Slinky at all levels utilized a software package written by a Turkish Engineering group (51) that allows sequential helical coiling of defined sized structures. This package requires a large workstation and Mathematica 12.3 (52). Mathematica was run on a Linux system with an Intel Core i7 CPU 2.80 GHz processor with four cores and on a Windows 7 system with an Intel Core i5 CPU 3.20 GHz processor with four cores. Output from both systems was displayed on a Samsung C27F591monitor and NVIDIA Quadro K1200 video card and 8 GB of memory. The various scripts for the software are supplied by the authors upon request. The digital files for the deconvolution and the movie data (Movies S1–S8) are deposited in the Electron Microscopy Databank (EMDB) database (), and detailed in *SI Appendix*.

### Computer Display Software.

Once structures are built, they are displayed in various dimensions, with the generalized display and quantitation software package written over the years by the Agard/Sedat groups (53). This software and its extensive Help files are available from the David Agard and J.S. UCSF emails. The displayed scripts are also available from the authors.

## Supplementary Material

Supplementary File

Supplementary File

Supplementary File

Supplementary File

Supplementary File

Supplementary File

Supplementary File

Supplementary File

Supplementary File

## Data Availability

[Tomographic] data have been deposited in the EMDB (https://www.ebi.ac.uk/emdb/) (EMD-14924, EMD-15057–EMD-15063). Further details appear in *SI Appendix*.
